# Reproductive biology, distribution and abundance of *Squalus megalops* (Macleay, 1881) and *Squalus mitsukurii* (Jordan & Snyder, 1903) off southern Brazil, southwest Atlantic

**DOI:** 10.1111/jfb.70080

**Published:** 2025-05-21

**Authors:** Mônica Camila Lourenço, Gabriel Canani, Maria Cristina Oddone

**Affiliations:** ^1^ Laboratório de Pesquisa em Chondrichthyes, Setor de Morfologia Instituto de Ciências Biológicas, Universidade Federal do Rio Grande – FURG Rio Grande Brazil; ^2^ Programa de Pós‐graduação em Oceanografia Biológica, Instituto de Oceanografia, Universidade Federal de Rio Grande – FURG Rio Grande Brazil; ^3^ Laboratório de Aves Aquáticas e Tartarugas Marinhas Instituto de Ciências Biológicas, Universidade Federal do Rio Grande – FURG Rio Grande Brazil; ^4^ Instituto Albatroz Rio Grande Brazil

**Keywords:** catch per unit effort (CPUE), dogfish sharks, reproductive parameters, sexual maturity, Squaliformes

## Abstract

*Squalus megalops* and *Squalus mitsukurii* are recognized as the most abundant *Squalus* species in the southern Brazil region. Currently, *S. megalops* is listed as ‘least concern’ (LC) and *S. mitsukurii* as ‘endangered’ (EN). However, at the regional level, both species are classified as ‘data deficient’ – DD, due to the lack of population local data. In this context, the present study aimed to determine the reproductive biology parameters of these species. In addition, insights on their abundance were provided. Research cruises using bottom trawls were conducted on the continental shelf off southern Brazil between 2001 and 2002. A total of 298 specimens of *S. megalops* (♂143|♀156) and 297 specimens of *S. mitsukurii* (♂129|♀166) were captured. For *S. megalops*, the L50 was 38.2 cm for males and 50.22 cm for females, with ovarian fecundity ranging from 1 to 4 vitellogenic follicles and uterine fecundity from 1 to 4 embryos. *S. mitsukurii* presented an L50 of 54.12 cm for males and 66.4 cm for females, with ovarian fecundity ranging from 1 to 8 vitellogenic follicles and uterine fecundity ranging from 1 to 8 embryos. The highest catch per unit of effort (CPUE) for *S. megalops* was in zone B (2360.43 ind/mn^2^) and for *S. mitsukurii* in zone A (5287.97 ind/mn^2^). The study observed differences in the reproductive stages and fecundity of both species compared to other studies for the same species. In southern Brazil, the calculated L50 sizes were smaller than those obtained in the northeast, potentially due to higher fishing pressure. Obtaining data on poorly known species reinforces the importance of monitoring fisheries in the region. The maturity data presented are crucial for species classified as DD, such as *S. megalop*s and *S. mitsukurii* in southern Brazil, potentially generating new information to support local fisheries management.

## 1 INTRODUCTION

The genus *Squalus* (Linnaeus, 1758) comprises small demersal sharks that can reach a total length of up to 1.5 m. These sharks are commonly found at depths between 50 and 1000 m, with some species such as *Squalus acanthias* typically found in shallower waters (50–150 m) (Compagno, 1984; Compagno et al., 2005; Ebert et al., 2021). They are characterized by the presence of a spine in each dorsal fin, the absence of an anal fin and well‐developed eyes (Compagno et al., 2005). Their distribution includes continental shelves, island slopes and submarine trenches of the Atlantic (including the Mediterranean), Pacific and Indian oceans (Compagno et al., 2005; Viana et al., 2016). Sex and age segregation behaviours have been identified among the species of the genus, and some species are considered to be resident, whereas others perform long‐distance migrations (Ebert et al., 2021).

Like other elasmobranchs, the life history of *Squalus* species is characterized by slow growth, late sexual maturation (average age of 15 years) and a long life expectancy, with individuals recorded at over 40 years (Bengil, 2022; Ebert et al., 2010, 2021) and up to 70 years in the case of *S. acanthias* (Linnaeus, 1758) (Cailliet et al., 2001). Gestation lasts up to 24 months, one of the longest recorded among vertebrates (Ebert et al., 2021; Natanson et al., 2017). The reproductive mode of *Squalus* is yolk‐sac viviparity, a strict form of lecithotrophic viviparity (Wourms, 1977), where the embryo develops in the uterus and obtains nutrients from the yolk stored in the yolk sac, which is directly connected to its digestive system (Walker, 2005). Musick and Ellis (2005) reported limited histotrophy in *Squalus* sharks, indicating that these species exhibit maternal investment that extends beyond strict lecithotrophy. In *Squalus megalops* and *Squalus mitsukurii*, vitellogenesis and gestation occur simultaneously (Calderón, 1994).

*S. megalops* (Macleay, 1881), commonly named short snout dogfish, occurs along the southern coast of Africa between the Gulf of Guinea and Mozambique (Atlantic and Indian Ocean), in the southern coast of Australia (Southern Ocean) and in the southeastern coast of Asia (Pacific Ocean) (Compagno, 1984). In Brazil, Soto (2001) reported that its distribution extends from Bahia (northeast Brazil) to Rio Grande do Sul (southern Brazil) in the south. In southern Brazil, specifically, *S. megalops* is the most common *Squalus* species, occurring at depths between 40 and 300 m (Calderón, 1994).

In southeastern Australia, the longevity of *S. megalops* has been estimated at 26 years for males and 32 years for females (Pajuelo et al., 2011). Other studies have examined various biological parameters of the species, including maturity parameters in South Africa (Bass et al., 1976; Watson & Smale, 1998), the maturity size of males and females in southeastern Australia (Braccini et al., 2006; Graham, 2005) and the size at first maturity and fecundity in northeastern Brazil (Hazin et al., 2006). Additionally, Calderón (1994) enhanced the understanding of *S. megalops* reproduction in southern Brazil, reporting on size at first maturity, ovarian and uterine fecundity and embryo size and weight at birth.

*S. mitsukurii* (Jordan and Snyder, 1903), commonly known as longnose dogfish, is distinguished by its round, long snout, a small spine on the dorsal fin and a dark band at the distal end of the caudal fin (Ebert et al., 2021; Last et al., 2007; Viana et al., 2016). The species was first described based on a specimen collected near Japan and is currently recognized as a Pacific inhabitant, with a distribution extending from the coast of North Korea to Vietnam in Southeast Asia (Finucci et al., 2020). However, specimens have also been recorded in various regions of the Atlantic, Indian and Pacific oceans, classified as either *S. mitsukurii* or *S*. cf. *mitsukurii* (Viana et al., 2016). These sharks are typically found at depths ranging from 100 to 700 m (Compagno et al., 2005).

Males of up to 23 years and females of up to 26 years have been recorded (Cotton et al., 2011). In southwest Atlantic, Oddone et al. (2010) reported maturity sizes in the Argentine‐Uruguayan Common Fishery Area. For northeastern Brazil, Fischer et al. (2006) estimated L50 values for both sexes. In southern Brazil, Calderón (1994) observed maturity sizes for males and females and also reported on ovarian and uterine fecundity, as well as embryo size and weight at birth.

Investigating the reproduction, distribution and population dynamics of sharks, such as *S. megalops* and *S. mitsukurii*, is essential for understanding their life histories and ecological significance (Cortés et al., 2012; Holden, 1974). For deep‐water species, this knowledge is critical for identifying vital areas like nurseries and migration routes, which are fundamental to developing effective conservation strategies and management frameworks. By addressing these key aspects, research on these sharks not only supports species‐specific management but also contributes significantly to preserving the balance and health of deep‐sea ecosystems. This comprehensive approach strengthens conservation efforts and promotes the sustainable use of fisheries resources within their habitats (Pajuelo et al., 2011). In Brazil, all knowledge of the catch composition of *S. megalops* and *S. mitsukurii* is based mainly on records from research fishing trips. The limited number of comprehensive studies on these species has resulted in a lack of robust regional population data, which has prevented accurate status classification for them. This is particularly concerning, as it is only the second study addressing the reproductive biology, distribution and abundance of these species in southern Brazil. The first study, conducted by Calderón (1994), remains unpublished, further highlighting the scarcity of available information.

On a global scale, *S. megalops* is listed as ‘least concern’ (LC) (Rigby & Kyne, 2020) in the International Union for the Conservation of Nature (IUCN) Red List of Threatened Species, whereas *S. mitsukurii* is listed as ‘endangered’ (EN) (Finucci et al., 2020). At the regional level, they are classified as ‘data deficient’ in the Biodiversity Extinction Risk Assessment System of the Chico Mendes Institute for Biodiversity Conservation (ICMBio, 2016). Therefore, our main goal was to assess the catch composition, reproductive biology and abundance of *S. megalops* and *S. mitsukurii* in southern Brazil, with a focus on conservation. As the data were collected 24 years ago, our results provide a historical baseline that can be useful for future assessments of population trends and potential conservation measures.

## 2 MATERIALS AND METHODS

### 2.1 Study area

**FIGURE 1:**
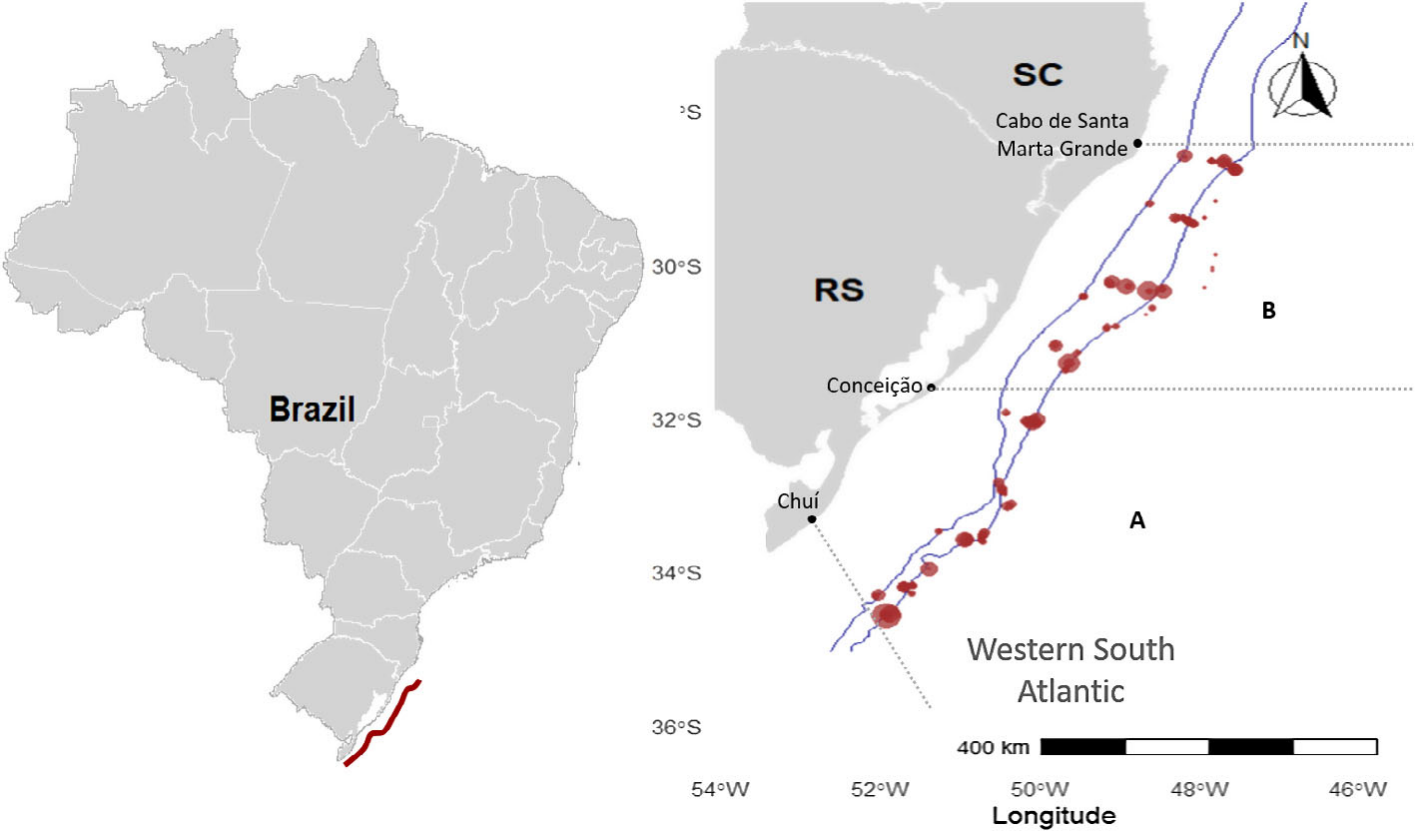
Study area and positions of fishing trawls conducted with bottom trawls in the southern region between Cabo de Santa Marta Grande and Chuí in 2001 and 2002. The dotted lines indicate the boundaries of the latitudinal layers divided between areas A and B.

The total area covered by the cruises in the Programa Nacional de Avaliação dos Recursos Vivos da Zona Econômica Exclusiva (REVIZEE)/Score Sul was divided into latitudinal bands, roughly corresponding to the latitudinal ranges covered by the scientific fishing cruise campaigns. The study area included two regions defined by the REVIZEE programme (Haimovici et al., 2008), extending from Chuí (Rio Grande do Sul state) to Cabo de Santa Marta Grande (Santa Catarina state). The area ‘A’ (southern part) was perpendicular to the coast from Chuí to 31°30′ S, south of Conceição (SC). The area ‘B’ (northern part) extended from 31°30′ S to 28°00′ S, north of Cabo de Santa Marta Grande (Figure 1).

The most significant oceanographic feature of this region is the subtropical convergence of the Brazil and Malvinas currents. The Brazil Current flows southward, characterized by warm, oligotrophic saline waters, whereas the Malvinas Current moves northward, transporting cold, less saline and nutrient‐rich waters (Castello & Moller Jr, 1997; Mendonça et al., 2017). This convergence leads to high biological productivity, making it an important fishing area (Perez et al., 2015).

Areas A and B (Figure 1), which are the focus of this study, exhibit oceanographic and ecological characteristics that may influence the distribution and abundance of *S. megalops* and *S. mitsukurii*. These regions are marked by intense dynamics of oceanic currents throughout the annual cycle (Gaeta & Brandini, 2006; Seeliger & Odebrecht, 2010), contributing to elevated marine productivity. This productivity can attract a variety of fish species and their predators, including the elasmobranch under investigation. Furthermore, these areas encompass a variety of complex marine habitats, such as estuaries, continental shelves, slopes and oceanic elevations. This ecosystem diversity provides favourable conditions for *S. megalops* and *S. mitsukurii*, offering both shelter and prey availability (Ivanoff et al., 2018; Vooren & Klippel, 2005).

### 2.2 Fisheries sampling

The samples were collected during six cruises of the REVIZEE/Score Sul with bottom trawls in the external borders of the continental shelf and upper continental slope of Rio Grande do Sul. The cruises were conducted in the winter of 2001 (August and September; hereafter ‘winter’) and the fall of 2002 (March and April; hereafter ‘fall’) on the research vessel ‘Atlântico Sul’, which is a 295‐t stern trawler, 35.9 m long, 8 m wide, 3.28 drafts, with a 860 HP MWM main engine (Table 1).

**TABLE 1:** Information on catches of the genus *Squalus* in the REVIZEE/Score Sul bottom trawl surveys conducted between Cabo de Santa Marta Grande (28°00′ S) and Chuí (34°34′ S) in 2001 and 2002.

Cruise	Year	Area	Season	Number of fishing trawls	Total swept area	Captures	
						*Squalus megalops ♂|♀*	*Squalus mitsukurii ♂|♀*
R00	2001	A	Winter	7	0.16 mn^2^	18|56	100|75
R01	2001	A	Winter	23	0.43 mn^2^	2|26	153|249
R02	2001	B	Winter	28	0.46 mn^2^	102|93	65|46
R07	2002	A	Fall	18	0.32 mn^2^	46|78	12|47
R08	2002	A	Fall	11	0.22 mn^2^	41|118	‐|5
R08	2002	B	Fall	13	0.24 mn^2^	104|108	‐|‐
R09	2002	B	Fall	15	0.32 mn^2^	81|50	41|32
**Total**				**115**		**394|529**	**371| 454**

The sampling depths varied between 100 and 600 m, with five defined depth ranges: 100–149, 150–199, 200–299, 300–399, 400–600 m. The bottom‐trawl net used was 439 meshes long, with mesh‐sizes of 160 mm near the opening and mesh‐sizes of 70 mm near the bag (end of the net). The lower net, measuring 40.4 m in length, was of the ‘Rockhopper’ type, with a 20.8 m central section fitted with 300‐, 200‐ and 130‐mm diameter rubber discs, and two 9.8 m side extensions, each fitted with 75‐mm‐diameter rubber discs. Most of the hauls took 30 min from the time the winch drum was locked to the start of the haul, and the hauling speed was 3 knots. The sampling station position was determined according to the suitability of the bottom to bottom trawl, with the use of the echosounder track line.

### 2.3 Biological sampling

The following biometric measurements were taken immediately after capture: total length (TL, cm) from the tip of the snout to the posterior end of the caudal fin and gonad weight (GW, g) were measured for each individual immediately after capture.

Maturity stages were classified as ‘immature’ and ‘mature’ on board based on macroscopic characteristics of the gonads and reproductive ducts for each individual. Female classification followed Fitz and Daiber (1963), recording gonad diameter (cm), the colour of the largest ovarian follicle (cm) and the number of vitellogenic follicles of the ‘maturing group’. Females were considered mature when large, yellow vitellogenic follicles were present in the ovary and embryos (free or candled, also known as polyvitelline capsules) were present in oviducts, following Peres and Vooren (1991) classification for lecithotrophic viviparous elasmobranchs. Polyvitelline capsules, or candled eggs, are reproductive structures in some yolk‐sac viviparous sharks. These ‘candle eggs’ contain multiple eggs or embryos, according to Sunye and Vooren (1997), within a single tertiary membrane, forming a unified reproductive unit (Hamlet et al., 2005). Numbers of mature preovulatory vitellogenic follicles and embryos were registered to determine ovarian and uterine fecundity, respectively. Due to the ovarian symmetry observed for both species, with two functional ovaries, the measurements were standardized using only values related to the right side. Male classification, in turn, followed Compagno, 1984, measuring clasper length from the point of insertion to the distal end, and manually classifying it as ‘rigid’ when the clasper offered resistance and did not bend easily and as ‘flexible’ when it showed the opposite characteristics.

Regarding species taxonomic classification, we have opted to retain the original group classifications (Compagno, 2005) for each species. Our decision to not use more recent taxonomic changes, as the ones described by Viana et al. (2016), was made due to the absence of access to all the sampled specimens for re‐identification.

### 2.4 Data analysis

The sex ratio was calculated, defined as the total number of females in relation to the total number of males (F:M). A χ^2^ test (Sokal and Rohlf, 1995) was used to test whether the calculated ratio for each species was significantly different from the expected 1:1. A significance level of 95% was considered for χ^2^ and the following tests.

Prior to following a parametric or non‐parametric analysis method, the Shapiro–Wilk test was used to determine if the data were normally distributed. The Mann–Whitney (MW) test (Sokal and Rohlf 1995) was used to examine differences in length distributions between zones A and B separately for immature and mature groups of each species. This test assesses whether one group tends to have larger values than the other, allowing us to infer potential differences in size structure between zones.

To analyse distribution and estimate abundance, catch per unit effort (CPUE) was calculated for each trawl conducted during each cruise as ind/mn^2^ by species and sex. The swept areas were obtained by multiplying the distances travelled, determined from the initial and final positions of each haul on the GPS, and the effective width of the nets. For comparing CPUE by sex among areas, a Kruskal–Wallis H‐test was used.

For analyses involving maturity, a total of 280 *S. megalops* (137 males and 143 females) and 210 *S. mitsukurii* (106 males and 104 females) had maturity records and were included in the following analyses. A binomial generalized linear model (GLM) (Nelder & Wedderburn, 1972) was used to analyse the relationship between the length of a population and its sexual maturity for each species separately. The logistic model:

(1) $$Y={\left[1+\exp\left\{-\left(a+\mathrm{bX}\right)\right\}\right]}^{-1}$$

(sensu Mollet et al., 2000) was fitted to the relationship between the proportion of mature individuals (*Y*) and TL classes of 10 cm (*X*). The parameters ‘*a*’ and ‘*b*’ were estimated using a binomial GLM. This model was used to estimate the lengths at which 50% (L50) and 90% (L90) of the population reached sexual maturity, calculated as:

(2) $$L_{50}=\frac{-a}{b}$$

and

(3) $$L_{90}=\frac{-\text{In}\left(1/9\right)-a}{b}$$

where ‘*a*’ is the intercept and ‘*b*’ is the coefficient of the TL in the GLM. The L50 represents the length at which half of the population is mature, whereas L90 indicates the length at which 90% of the population has reached maturity, providing a more comprehensive understanding of the maturation process within the population. For statistical analysis, the maturity states were transformed into a binomial dataset: immature = 0, mature = 1.

Statistical analyses were performed using the free software R, version 4.3.3 (R Development Core Team, 2024).

### 2.5 Ethical considerations

This research is part of the project titled ‘The Biology and Conservation of Shark Populations in the Extreme South of Brazil’, registered at the Universidade Federal do Rio Grande under process number 814440/2014. The study was conducted with the approval of the Ethics Committee on Animal Use (Comissão de Ética Em Uso Animal – CEUA) of the same university. No experimental work was undertaken with the collected specimens.

## 3 RESULTS

### 3.1 Catches composition

*S. megalops* was represented by 298 individuals and *S. mitsukurii* by 297. For both species, a total of 271 males and 322 females were recorded, in which 161 were immature and 319 were mature. The sex ratio of *S. megalops* was 1:0.9, not a statistically significant difference from the expected 1:1 ratio (*χ* ^2^ = 0.66, *p* = 0.42), whereas *S. mitsukurii* was 1:0.7, with a significant statistical difference slightly favouring females (*χ* ^2^ = 4.64, *p* = 0.03). The frequency distributions of TL were not normal for both species (*p* < 0.05). Total length frequency distributions showed that immature individuals occurred more frequently for the females of both species, whereas the catch of the males was mostly composed of mature individuals.

**FIGURE 2:**
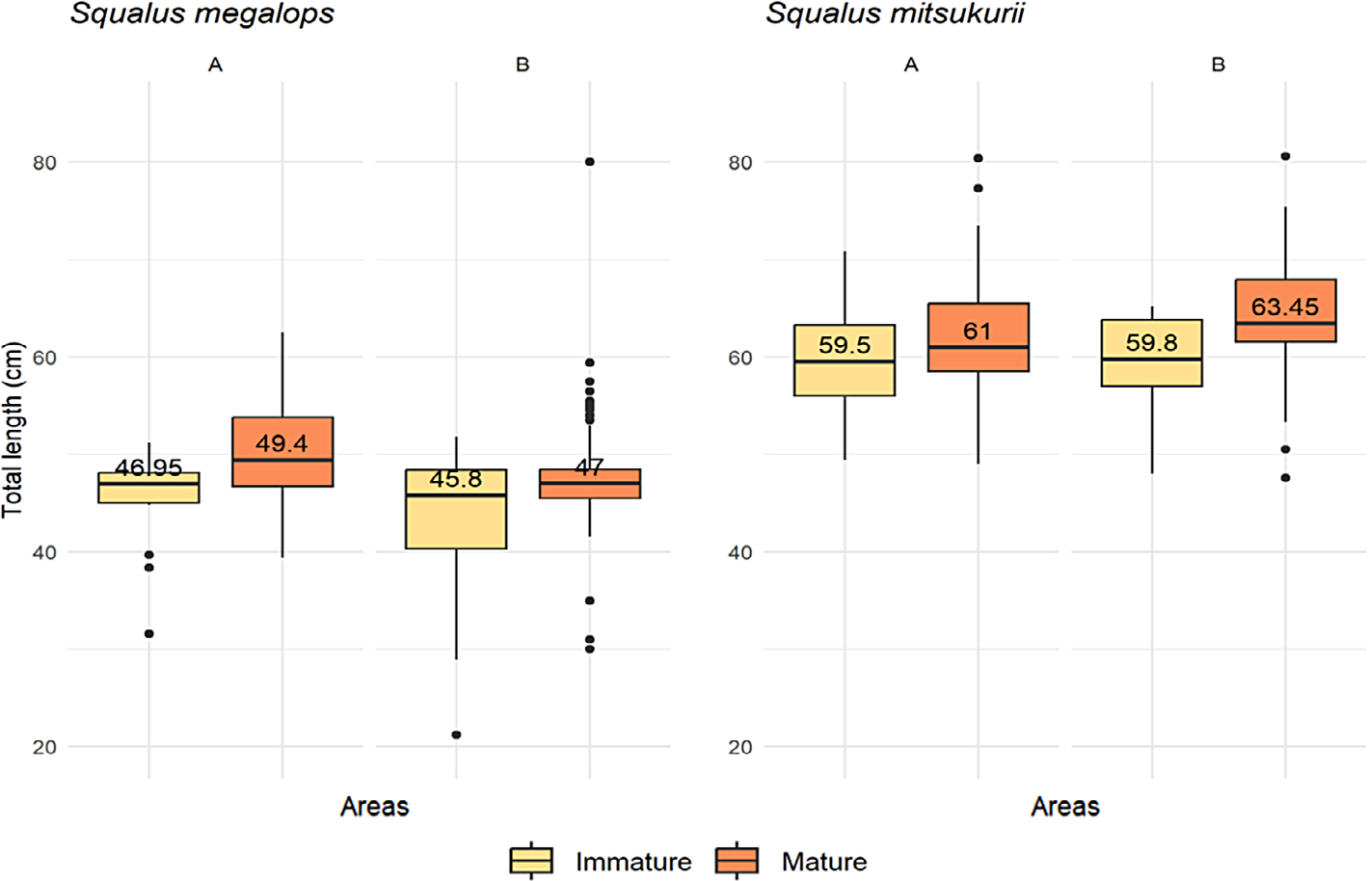
Box plot showing the total length distribution of immature and mature individuals of *Squalus megalops* and *Squalus mitsukurii* in areas A and B, based on bottom‐trawling surveys conducted during the REVIZEE/Score Sul project in 2001 and 2002 between Cabo de Santa Marta Grande (28°00′ S) and Chuí (34°34′ S). The line in the centre of each box represents the median value.

Significant differences in TL were found between areas and maturity stages. For *S. megalops*, there was no significant difference between areas A and B for immature individuals (*Z* = 1.13, *p* = 0.25). However, for mature individuals, there was a significant difference between areas (*Z* = 4.26, *p* = 1.99e‐05), indicating that larger individuals are predominantly located in area A, based on the comparison of medians (Figure 2). For immature *S. mitsukurii* individuals, the results also showed no significant difference between areas A and B (*Z* = −0.29, *p* = 0.76). Nevertheless, for mature individuals, a significant difference between areas was observed (*Z* = −2.68, *p* = 0.007), with larger individuals being predominantly found in area B, contrary to the observation for *S. megalops* (Figure 2).

*S. megalops* was represented by 142 males, with TLs ranging from 29.5 to 62.5 cm. Of these, 15 (11%) were classified as immature and 122 (89%) as mature. A total of 156 females were collected with TLs ranging from 21.2 to 80.0 cm. Of these, 76 (53%) were immature, and the remaining 67 (47%) were mature.

For *S. mitsukurii*, 131 males ranging from 47.6 to 70.5 cm TL were recorded. Of these, 5 (5%) were immature, and 101 (95%) were mature. A total of 166 females were collected, measuring between 48.0 and 80.9 cm TL, of which 40 (38%) were immature and 64 (62%) were mature.

### 3.2 Reproductive biology

**FIGURE 3:**
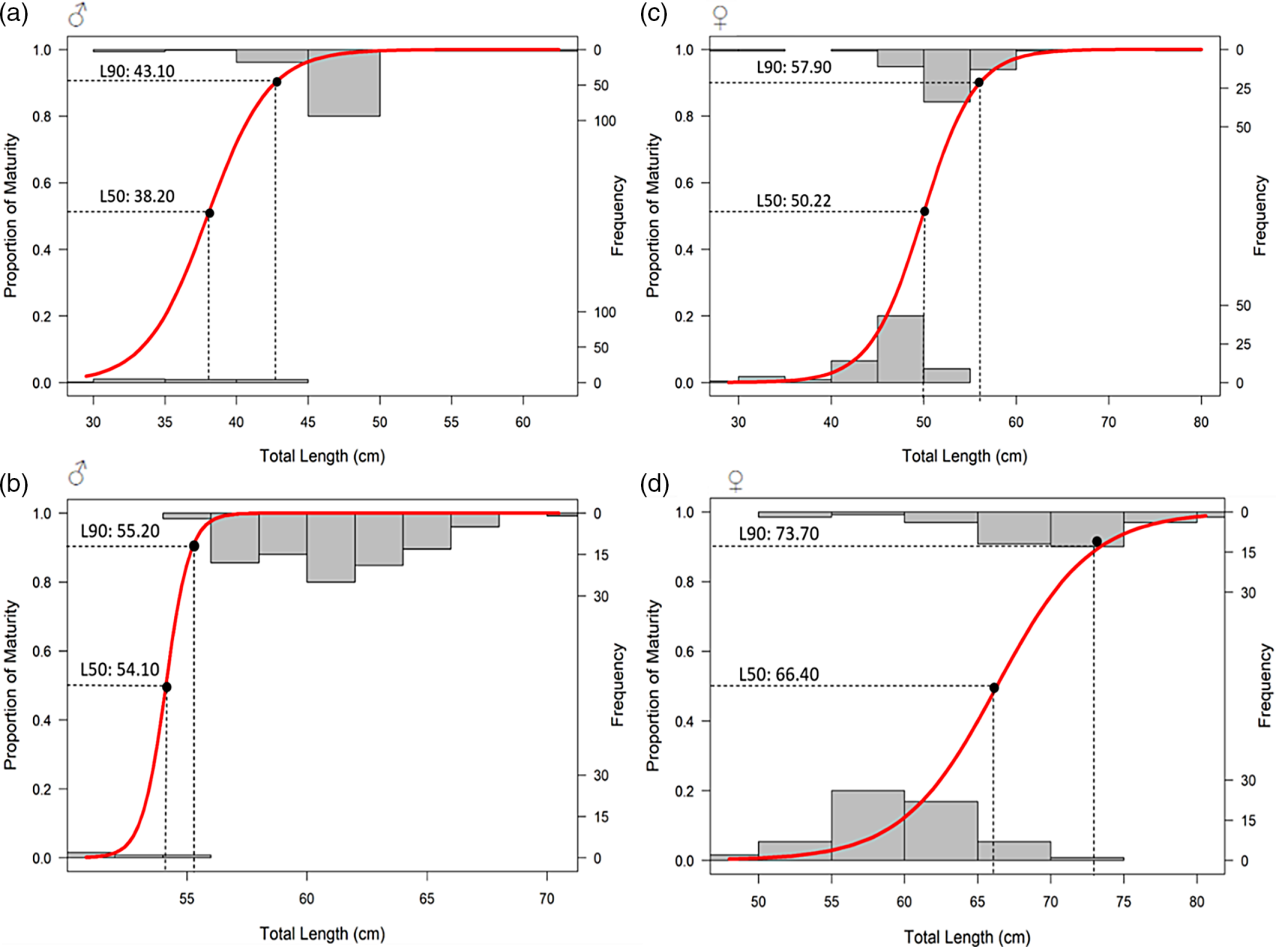
Maturity ogive for females of *Squalus megalops* (a – males, c – females) and *Squalus mitsukurii* (b – males, d – females) from the REVIZEE/Score Sul bottom trawl surveys conducted in 2001 and 2002 between Cabo de Santa Marta Grande (28°00′ S) and Chuí (34°34′ S). The histogram represents the frequency of the size classes of individuals in the sample (right *Y* axis), whereas the red curve represents the proportion of mature females calculated in relation to the total length classes of 10 cm (left *Y* axis).

For *S. megalops*, clasper length was recorded in four males, ranging from 3.6 to 4.0 cm. The maturity ogive analysis estimated an L50 of 38.2 cm and an L90 of 43.1 cm for males (Figure 3a). In females, these values were 50.2 and 57.9 cm, respectively (Figure 3c). For *S. mitsukurii*, 16 mature males had claspers measuring between 5.1 and 5.8 cm. The L50 and L90 were estimated at 54.1 and 55.2 cm TL (Figure 3b), whereas in females, these thresholds were 66.4 and 73.7 cm TL (Figure 3d).

**FIGURE 4:**
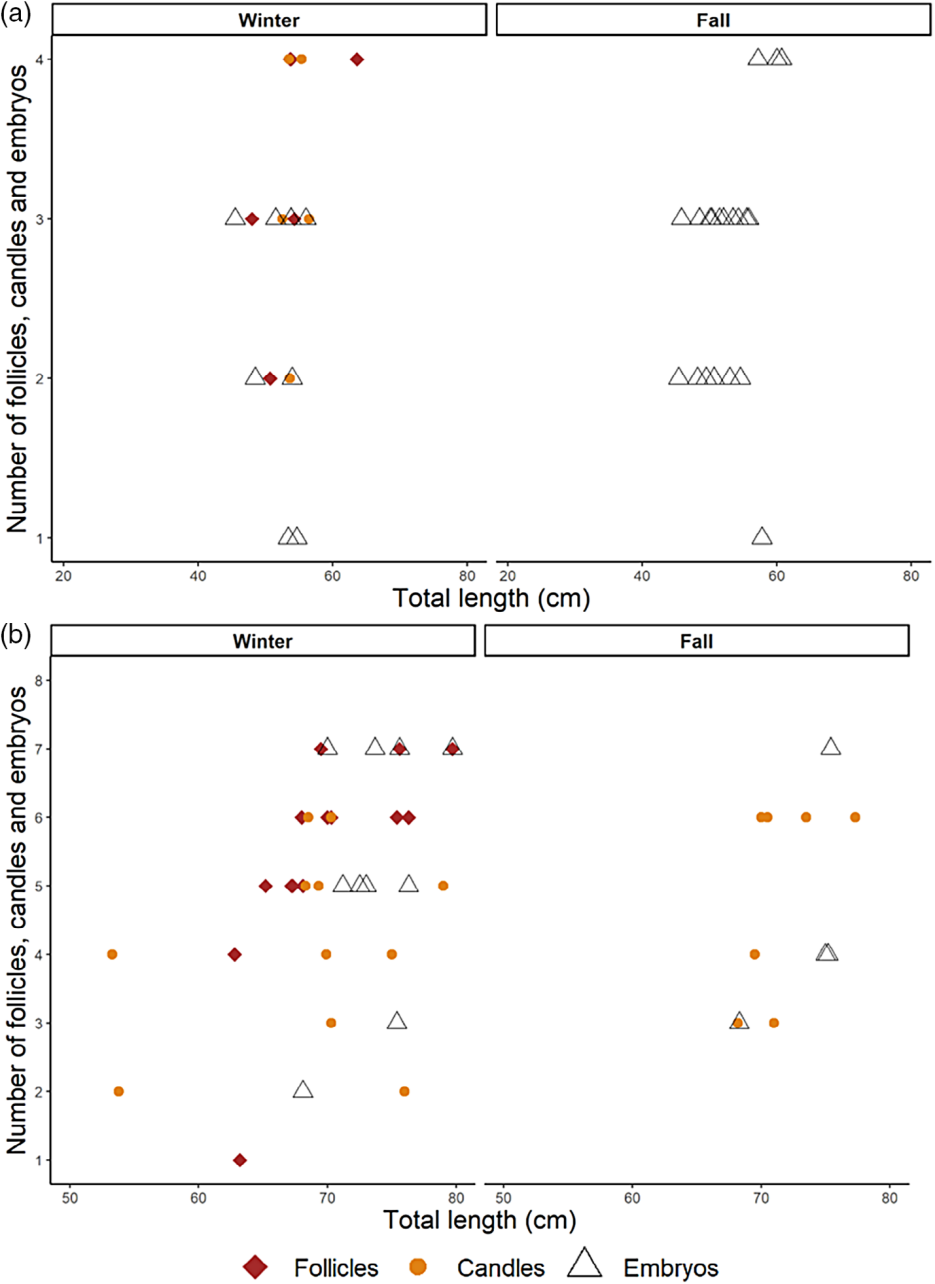
Relationship between total female length and uterine and ovarian fecundity for females of (a) *Squalus megalops* and (b) *Squalus mitsukurii* caught in the REVIZEE/Score Sul bottom trawl surveys carried out in 2001 and 2002 between Cabo de Santa Marta Grande (28°00′ S) and Chuí (34°34′ S).

For *S. megalops*, ovarian fecundity (*n* = 6) ranged from 1 to 4 vitellogenic follicles, and uterine fecundity (*n* = 34) varied from 1 to 4 embryos. Candled eggs in the uterus were observed in five females (Figure 4a). A total of 42 pregnant females were recorded, with 29 individuals found in area A and 13 in area B. The largest immature female measured 51.8 cm TL, whereas the smallest mature one was a 30.0 cm TL pregnant female.

For *S. mitsukurii*, ovarian fecundity (*n* = 14) ranged from 1 to 8 vitellogenic follicles, whereas uterine fecundity (*n* = 34) varied from 1 to 8 embryos. Candled eggs in the uterus were observed in eight females (Figure 4b). In total, 36 pregnant females were recorded, with 24 individuals found in area A and 12 in area B. The largest immature female measured 70.8 cm TL, whereas the smallest mature female was a pregnant individual, measuring 53.3 cm TL. A total of 19 full‐term embryos were sampled during the winter season, with TLs ranging from 19.9 to 23 cm. According to observational notes recorded in the database, these embryos exhibited respiratory activity but lacked swimming mobility.

### 3.3 Abundance

**FIGURE 5:**
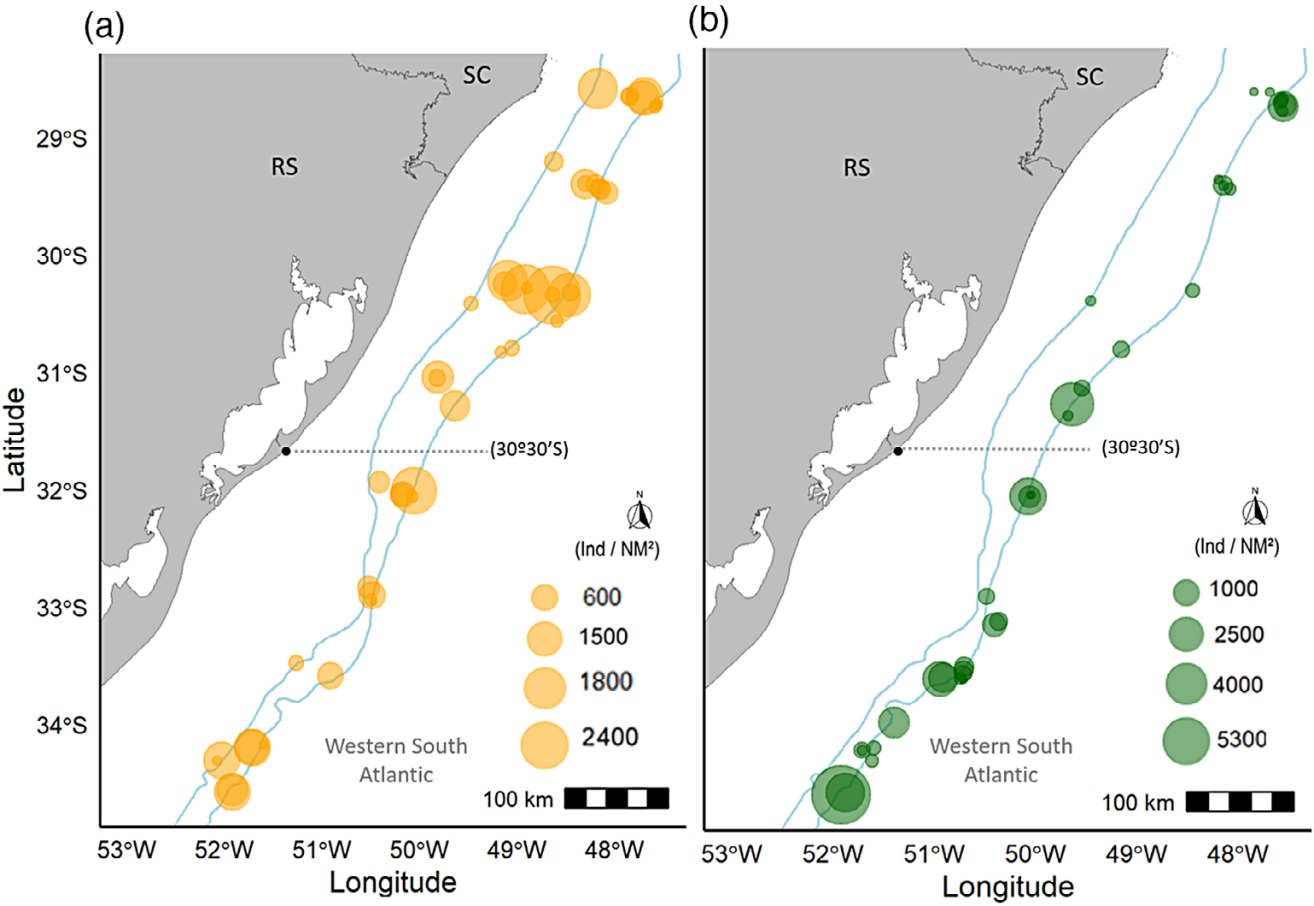
Catch per unit effort of (a) *Squalus megalops* and (b) *Squalus mitsukurii* from REVIZEE/Score Sul by bottom trawl surveys conducted in 2001 and 2002 between Cabo de Santa Marta Grande – SC (28°00′ S) and Chuí – RS (34°34′ S). The dashed line (30°30′ S) indicates the division between area A (southern part) and area B (northern part).

In relation to the CPUE, the highest value among the *Squalus* was recorded for *S. mituskurii*, with 5287.97 ind/mn^2^ for area A, whereas the highest value for *S. megalops* was 2360.43 ind/mn^2^, reported for area B (Figure 5).

**FIGURE 6:**
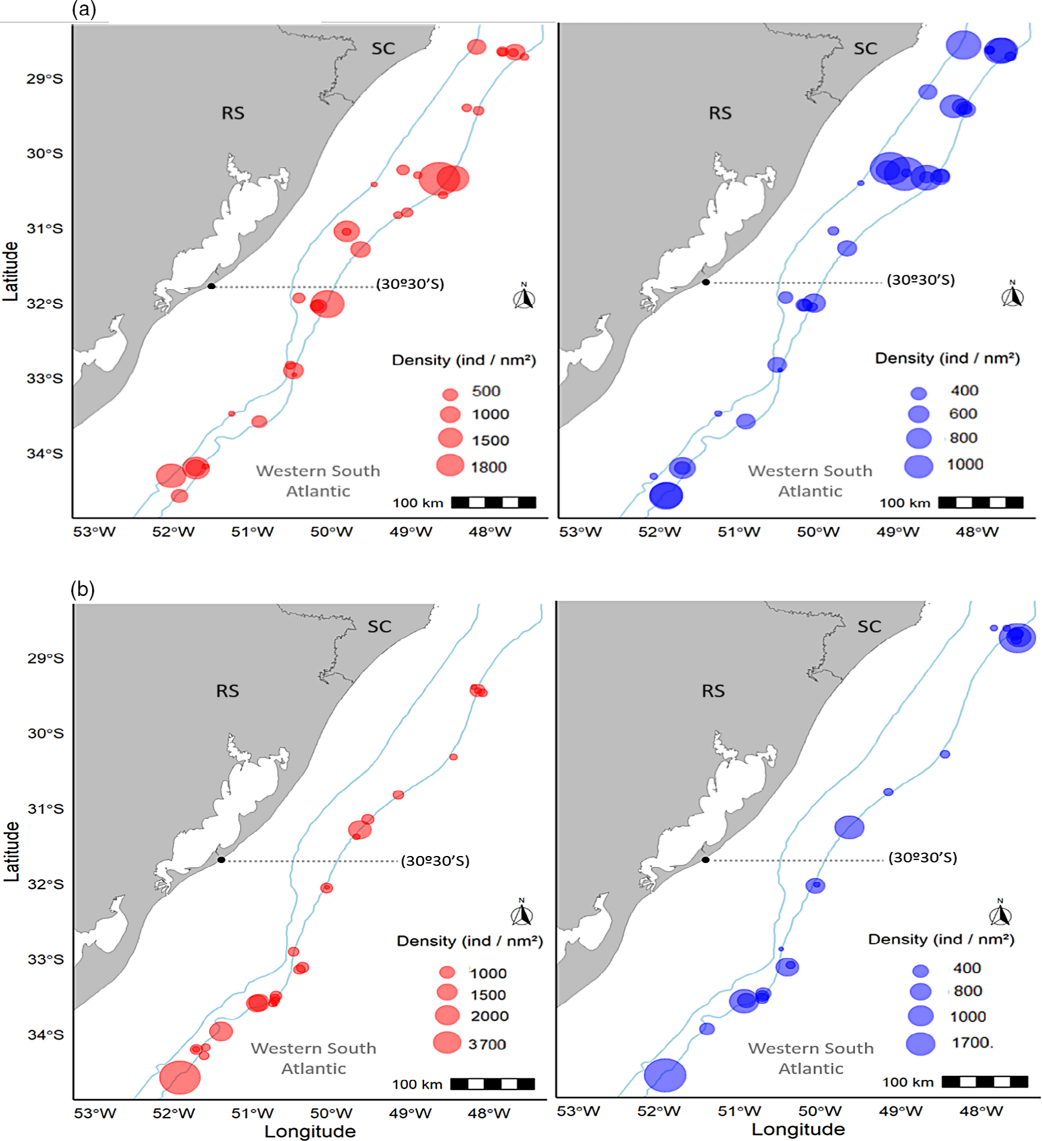
Catch per unit effort of (a) *Squalus megalops* and (b) *Squalus mitsukurii* by sex (male = blue circle; female = pink circle) from REVIZEE/Score Sul by bottom trawl surveys conducted in 2001 and 2002 between Cabo de Santa Marta Grande – SC (28°00′ S) and Chuí – RS (34°34′ S). The dashed line (30°30′ S) indicates the division between area A (southern part) and area B (northern part).

Female CPUE of *S. megalops* showed a wider range of abundance than males, with values ranging from a minimum of 52.51 ind/mn^2^ to a maximum of 1801.38 ind/mn^2^ in a single haul. Males showed a range between 51.97 and 1000.36 ind/mn^2^ per haul. The highest numbers of both sexes were recorded in area B. No statistically significant differences in CPUE were found between areas for either females (*K* = 0.78, *p* = 0.38) or males (*K* = 3.36, *p* = 0.07) (Figure 6a).

Female CPUE of *S. mitsukurii* showed a wider range of abundance than males, with values ranging from 53.61 to 3654.92 ind/mn^2^ in a single haul. Males showed a range between 51.67 and 1633.05 ind/mn^2^ per haul. The highest numbers for both sexes were recorded in area A. A statistically significant difference in CPUE between areas was found for females (*K* = 4.11, *p* = 0.04) but not for males (*K* = 0.21, *p* = 0.64) (Figure 6b).

## 4 DISCUSSION

Among the elasmobranchs caught in the bottom trawls of the REVIZEE programme, *S. megalops* was the fourth most abundant species by weight, whereas *S. mitsukurii* ranked third. These two species have also been recognized as the most abundant *Squalus* species in southern Brazil (Haimovici et al., 2008). According to Calderón (1994), *S. megalops* would be a constantly present species in the studied area, whereas *S. mitsukurii* along with *S. acanthias* would occur sporadically. The constant presence of mature males suggests a non‐synchronized individual sexual cycle at the population level, as observed by Calderón (1994), indicating availability for copulation throughout the year, which was also observed by Oddone et al. (2010) for *S. mitsukurii* in the Argentine‐Uruguayan Common Fishery Area.

A predominance of mature males was observed for both species, whereas females showed no clear pattern of dominance. These findings suggest a potential spatial (latitudinal) segregation based on maturity between sexes across both species, with significant implications for life‐history strategies and population dynamics. This differential distribution pattern may be attributed to specific reproductive behaviours or habitat preferences associated with various life stages. The female reproductive cycle of both species seems asynchronous within the population, as indicated by the simultaneous occurrence of various stages of gestation (Calderón, 1994; Pajuelo et al., 2011). Our study supports this observation by finding females that had both developing follicles and either eggs with visible embryos or fully formed embryos. The discovery of females carrying embryos close to birth suggests the possible presence of a nursery area, but additional research is needed to confirm this hypothesis (Table 2).

The observed sex ratio for *S. megalops* was 1:1, which contrasts with findings from other studies (e.g., Braccini et al., 2006; Graham, 2005; Hazin et al., 2006; Watson & Smale, 1998) that reported different sex ratios in other regions. These discrepancies may be influenced by methodological differences, such as sampling techniques and spatial coverage. For instance, Hazin et al. (2006) used baited traps and bottom longlines, whereas Braccini et al. (2006) used commercial shark fishing vessels with gillnets. Although the fishing gear used in our study (bottom trawl) was the same as in the studies by Graham (2005) and Watson and Smale (1998), the geographical areas of their research differed – southeastern Australia and South Africa, respectively – and this may have potentially influenced the sex ratio outcomes.

**TABLE 2:** Total number (TN) and percentage (%) of analysed specimens of *Squalus megalops* and *Squalus mitsukurii* by area and sex.

	*Squalus megalops* (*n* = 298)				*Squalus mitsukurii* (*n* = 297)			
	Area				Area			
	A (*n* = 115)		B (*n* = 183)		A (*n* = 153)		B (*n* = 72)	
	♂	♀	♂	♀	♂	♀	♂	♀
TN (%)	44 (38%)	71 (62%)	98 (54%)	85 (46%)	65 (42%)	88 (58%)	41 (57%)	31 (43%)
Mean TL ± SD	47.0 ± 5.1	51.2 ± 4.3	44.9 ± 4.3	47.7 ± 8.1	59.4 ± 3.7	63.3 ± 7.2	62.0 ± 3.7	65.1 ± 8.0
Median TL	46.6	51	46.4	48.3	59.3	61.5	62.6	64.4
p25	45.4	48	44.1	45	57.4	57.5	60.9	59.1
p75	47.5	53.9	47.5	51.5	61.4	68.7	63.8	70.45
Immat	2	18	13	57	5	52	0	14
Mat	38	43	84	24	60	36	41	17
Median immat	36.6	47.1	37.3	46.6	51.5	59.8	‐	59.0
Median mat	46.8	53.4	46.6	52.6	59.8	70.5	62.6	69.9
p25 immat	33.6	46	33.5	44.2	50.8	57.1	‐	57.0
p75 immat	37.6	48.3	40.4	49	53.1	63.9	‐	63.8
p25 mat	45.5	50.2	45.1	49.6	57.9	68.1	60.9	68
p75 mat	48.0	54.7	47.7	55.1	61.5	75.1	63.7	75

*Note*: Mean total length (mean TL) with standard deviation (SD in parenthesis), median TL and 25th and 75th percentiles (p25, p75). Number (immat, mat) of immature and mature individuals are included. Median TL and 25th and 75th percentiles for immature (median immature, p25 immat, p75 immat) and mature (median mat, p25 mat, p75 mat) individuals.

For *S. mitsukurii*, the sex ratio showed a significant bias in favour of females. Similar patterns have been reported by Fischer et al. (2006) in the southwestern equatorial Atlantic and Litvinov (1990) in the southeastern Pacific, both of whom observed a significant female bias. Such differences in sex ratio are common in several *Squalus* species (Demirhan et al., 2007). Furthermore, the disparity in CPUE abundance between females and males observed in this study (Table 3) supports these findings and may be related to sexual segregation.

**TABLE 3:** Reproductive parameters estimated for *Squalus megalops* and *Squalus mitsukurii* from REVIZEE/Score Sul by bottom trawl surveys conducted between Cabo de Santa Marta Grande (28°00′ S) and Chuí (34°34′ S) in 2001 and 2002 and those reported by other authors elsewhere.

*Squalus megalops*							
Locality	Sex ratio *♂|♀*	L50*♂*	L50*♀*	OF	UF	Size at birth	Reference
Southern Brazil	‐	40.0	46.0	1–5	1–4	20.0–22.0	Calderón (1994)
South Africa	‐	40.0	50.0	‐	1–4	23.2–27.7	Watson and Smale (1998)
Northeastern Brazil	1:5.6	43.0–46.0	59.0	1–8	1–8	20.0–23.0	Hazin et al. (2006)
Southeastern Australia	‐	37.3–39.8	45.9–49.5	‐	‐	19.1–24.4	Braccini et al. (2006)
Central‐eastern Atlantic Ocean	1:2.6	49.0	63.4–65.9	1–6	1–6	22.6	Pajuelo et al. (2011)
Southern Brazil	0.9:1	38.20	50.22	1–4	1–4	‐	This study

*Squalus mitsukurii*							
Locality	Sex ratio *♂|♀*	L50*♂*	L50*♀*	OF	UF	Size at birth	Reference
Southern Brazil	‐	54.0	65.0	1–9	2–9	23.0–24.0	Calderón (1994)
Northeastern Brazil	1:4.6	65.0	77.8	3–27	3–11	‐	Fischer et al. (2006)
Southwestern Atlantic Ocean	1:1	55.9	43.1	1–15	1–10	22.0–24.0	Oddone et al. (2010)
Middle of the Pacific Ocean	‐	47.0	64.0	‐	3–10	23.3	Cotton et al. (2011)
Southern Brazil	0.7:1	54.12	66.4	1–8	1–8	‐	This study

Abbreviations: OF, ovarian fecundity; UF, uterine fecundity.

The geographic and bathymetric distribution of *S. megalops* and *S. mitsukurii* off the southern coast of Brazil showed distinct patterns, reflecting their specific ecological and behavioural adaptations. For instance, *S. megalops* was observed throughout the study area (areas A and B), with a tendency of higher catches in area B. Calderón (1994) observed that the species occurs at depths between 40 and 300 m, being more frequent in catches during the winter and spring months, when its density is high at bottom temperatures of 12 to 17°C. On the contrary, *S. mitsukurii* showed a more restricted geographical distribution, occurring more frequently to the south of Cabo de Santa Marta Grande, with a tendency to higher catches in area A.

Vooren (1997) classified *S. megalops* as a migratory species that follows the subtropical convergence zone formed by the Brazil Current and the Falklands Current. Although its occurrence is reported throughout the year on the Rio Grande do Sul shelf, its abundance is higher in winter due to a higher influence of the Falklands Current, which displaces the convergence zone further north over the Brazilian coast. Conversely, *S. mitsukurii* is a winter migrant in southern Brazil (Vooren, 1997), reproducing in Uruguayan and Argentinean waters in summer and returning to the continental shelf of southern Brazil in winter. Calderón (1994) observed *S. mitsukurii* occupying the southernmost areas (area A), noting that the species was absent in the study area during summer, present in high densities in winter and found in the northern part of the study area in fall. This suggests that *S. mitsukurii* migrates with the Falklands Current, as observed by Menni and Lopez (1984) in Argentina.

Significant differences in TL were found between mature individuals in areas A and B for both *Squalus* species. *S. megalops* tended to have larger individuals in area A, whereas *S. mitsukurii* had larger individuals in area B. There were significant differences between the two species in their respective TL ranges. The smaller specimen of *S. megalops* in record was 21.20 cm TL, whereas for *S. mitsukurii*, it was 40 cm TL. Given the estimated birth size of approximately 22 cm TL described by Hazin et al. (2006), this smallest individual was probably a neonate or in its first year of life. Size and sex segregation are well‐documented phenomena among *Squalus* species, although population structures may exhibit variability across species and geographic locations (Braccini et al., 2006; Compagno et al., 2005; Ebert, 2013; Graham, 2005; Pajuelo et al., 2011).

Females of both species reached sexual maturity at larger sizes than males. This has been documented in previous studies for *S. megalops* (Bass et al., 1976; Braccini et al., 2006; Calderón, 1994; Graham, 2005; Hazin et al., 2006) and *S. mitsukurii* (Calderón, 1994; Cotton et al., 2011; Fischer et al., 2006; Oddone et al., 2010; Watson & Smale, 1998; Wilson & Seki, 1994) from other parts of the world, confirming the sexual size dimorphism in this species in many localities. This pattern is consistent with most viviparous species of sharks, as females need a large size at maturity because of their energetically demanding reproductive mode (Sims, 2003).

Our findings corroborate the trend of relatively smaller sizes observed in the southern region of Brazil, which may be attributed to two hypotheses: the existence of distinct populations or differential levels of exploitation. The significantly higher fishing mortality in southern waters may have induced a compensatory reduction in the size at first maturity, as these stocks have been subjected to trawling for several decades (Vooren et al., 1990). Conversely, the *Squalus* populations in northeastern Brazil have remained largely unexploited (Fischer et al., 2006), potentially explaining the observed size differences. This disparity in exploitation history between the two regions provides a unique opportunity to examine the effects of fishing pressure on life‐history parameters.

Hazin et al. (2006) argue that historically intense fishing activities off southern Brazil may have triggered a density‐dependent response in female *S. megalops*, resulting in smaller size at maturity compared to their northeastern counterparts. This hypothesis is corroborated by the observation that the disparity in size at maturity between northeastern and southern populations is more pronounced in females than in males. Furthermore, this phenomenon is consistent with the species reproductive mode, which inherently requires females to attain a larger size.

Previous studies on *S. acanthias* have demonstrated how changes in stock abundance can influence the size at first maturity of individuals (Sosebee, 2005). This research suggests that during periods of high stock abundance, the size at which 50% of the population reaches sexual maturity (L50) increases, whereas periods of intense fishing pressure that reduce the biomass of adult females result in a decline in the size at first maturity. Comparing our maturity ogive results with those described by Calderón (1994) for the same region 15 years earlier, we observed different patterns between species and sexes. For *S. megalops*, females in our study had a higher L50 compared to Calderón's results, whereas males had a slightly lower L50. For *S. mitsukurii*, both sexes showed similar L50 values to those reported by Calderón. This observation may suggest a slight recovery from intense fishing in the southwest Atlantic, but it is limited to a small sampling. However, the CPUE observed by Calderón (1994) during the period 1980–1987 and by Haimovici et al. (2008) during 2001–2002 on the continental shelf of Rio Grande do Sul did not detect any significant increase in abundance.

The ovarian fecundity of *S. megalops* ranged from 1 to 4 vitellogenic follicles, whereas the uterine fecundity varied between 1 and 4 embryos, closely aligning with Calderón's (1994) findings and falling within the range reported by Hazin et al. (2006) in the northeast (Table 3). For *S. mitsukurii*, fecundity ranged from 1 to 8 vitellogenic follicles and embryos, which is consistent with the ranges previously documented by Oddone et al. (2010) (1–15 follicles and 1–10 embryos) and Calderón (1994) (1–9 follicles and 2–9 embryos). Contrary to Calderón's (1994) observations, which reported the smallest pregnant females at 45 and 65 cm for *S. megalops* and *S. mitsukurii*, respectively, our study documented pregnant females at considerably smaller sizes: 30 cm for *S. megalops* and 53.3 cm for *S. mitsukurii*. This discrepancy provides evidence that females may be experiencing greater reproductive impacts from fishing pressure.

The reproductive cycles of *S. megalops* and *S. mitsukurii* exhibited distinct seasonal patterns. In *S. megalops*, a comprehensive range of reproductive stages was observed during winter, including vitellogenic follicles, candle and embryos. However, only embryos were recorded in fall, suggesting a potential seasonal shift in reproductive activity. In contrast, female *S. mitsukurii* were documented during winter with vitellogenic follicles, candle and embryos, indicating ongoing reproductive processes. In fall, candle and embryos were observed, suggesting a potentially extended or continuous reproductive cycle in this species. Nevertheless, definitive conclusions cannot be drawn due to uncertainties in recording of vitellogenic follicle data by the sampler.

Complex asynchronous cycles have been reported for *Squalus* species (Cotton et al., 2011; Watson & Smale, 1998; Yano, 1995), making it challenging to validate a definitive reproductive cycle due to insufficient temporal replication. To properly investigate reproductive seasonality, it would be necessary to conduct seasonal sampling over an extended period, spanning several years.

The simultaneous occurrence of vitellogenesis and gestation in *S. mitsukurii* and *S. megalops* was previously observed and documented (Sala‐y‐Gomez seamounts: Litvinov, 1990; southern Brazil: Calderón, 1994; southwestern equatorial Atlantic Ocean: Fischer et al., 2006). Oddone et al. (2010) suggest that birth of *S. mitsukurii* may occur between fall and winter due to the high TL class of the embryos (22 cm), which was also found for *S. mitsukurii* in the present study, and that ovulation may occur immediately after birth (Figure 4).

A recent assessment of scientific progress in the biology of marine elasmobranchs in Brazil (as listed in MMA Ordinance no. 445/2014) evaluated species‐specific studies conducted between 2015 and 2020 (Kotas et al., 2023). *S. acanthias*, a representative of the Squalidae family, was classified as having made ‘no progress’ in the research categories of ‘distribution areas’ and ‘reproductive biology’. The most thoroughly studied species in its genus has shown only limited scientific progress. This makes the situation for *S. megalops* and *S. mitsukurii* even more concerning.

The current lack of knowledge has led to their data deficient status, emphasizing the need for targeted research to understand their distribution, population dynamics and conservation status. Such research is crucial for developing effective management and conservation strategies for these potentially vulnerable species. As a result, this study, despite relying on short temporal sampling historical data, can provide a valuable baseline for tracking future changes in the population and biology of *Squalus* species in the area, which is important for its conservation planning and management.

It is important to note that the CPUE data are from more than 20 years ago, which means that the abundance of *S. megalops* and *S. mitsukurii* in the region may have changed significantly over this period. Changes in fishing dynamics and fishing pressure may have affected the populations of these species. Therefore, updated and continuous studies are essential to reassess the current situation and ensure proper management of the populations, especially in the face of possible changes in ecological conditions and exploitation levels.

Our study contributes to the understanding of the reproductive biology and population dynamics of *S. megalops* and *S. mitsukurii* in southern Brazilian waters. However, it is important to highlight that the data were collected more than two decades ago, so they might not reflect the current status of these populations. The sampling showed spatial segregation for maturity stages in males but not for females. These findings provide essential insights into the reproductive parameters for the studied species, suggesting the study area as a potential nursery area (presence of pregnant female) and subsidizing a growing body of knowledge crucial for conservation and management towards more sustainable fisheries.

## AUTHOR CONTRIBUTIONS

**Monica Lourenço:** formal analysis (equal), investigation (lead), methodology (equal), software (equal), writing – original draft (lead), writing – review and editing (equal). **Gabriel Canani:** software (equal), formal analysis (supporting), methodology (supporting), writing – (supporting). **Maria Cristina Oddone:** conceptualization (lead), data curation (lead), formal analysis (equal), methodology (lead), project administration (lead), supervision (lead), writing – review and editing (equal).

## FUNDING INFORMATION

This study was funded by the Conselho Nacional de Desenvolvimento Científico e Tecnológico.
